# Targeted therapy for metastatic renal cell carcinoma

**DOI:** 10.1038/sj.bjc.6602978

**Published:** 2006-02-07

**Authors:** P H Patel, R S K Chaganti, R J Motzer

**Affiliations:** 1 Department of Medicine, Memorial Sloan-Kettering Cancer Center, 1275 York Avenue, New York, NY 10021, USA; 2 Cell Biology Program, Memorial Sloan-Kettering Cancer Center, 1275 York Avenue, New York, NY 10021, USA

**Keywords:** von Hippel–Lindau, sunitinib, sorafenib, CCI-779, bevacizumab, erlotinib

## Abstract

Metastatic renal cell carcinoma (RCC) has historically been refractory to cytotoxic and hormonal agents; only interleukin 2 and interferon alpha provide response in a minority of patients. We reviewed RCC biology and explored the ways in which this understanding led to development of novel, effective targeted therapies. Small molecule tyrosine kinase inhibitors, monoclonal antibodies and novel agents are all being studied, and phase II studies show promising activity of sunitinib, sorafenib and bevacizumab. The results of phase III studies will determine the role of these agents in metastatic RCC.

Malignant renal cell carcinoma (RCC) accounts for 2–3% of cancer incidence and results in over 100 000 worldwide deaths annually. In developed nations the average age-adjusted incidence of RCC is approximately 12/100 000 in men and 5/100 000 in women. RCC is the most lethal urologic cancer and the sixth leading cause of cancer deaths in the developed nations. For reasons that are unclear, RCC age-adjusted incidence has been rising for the past 30 years within the US and most European nations at an annual rate of approximately 3% (Chow *et al*, 1999). For those who present with metastasis, the overall clinical course of RCC varies; approximately 50% of patients survive <1 year and 10% survive for over 5 years (Motzer *et al*, 2004). For those who present with early stage disease, radical nephrectomy is the treatment of choice; however, 30% of these patients will relapse and develop future metastasis. Chemotherapy has consistently been an ineffective form of treatment for RCC (Motzer *et al*, 1996), and until recently, the only effective treatment for metastatic disease was cytokine-based immunotherapy with interferon (IFN)-*α* or interleukin (IL)-2, which have a response rate of approximately 15% (Rosenberg *et al*, 1987). Recent advances in understanding the biology and genetics of RCC have led to several novel targeted approaches for the treatment of metastatic RCC, with higher response rates.

## RCC GENETICS

Consistent with the multistep carcinogenesis model, RCC is thought to arise from a complex set of events during which the tumour cells encounter a variety of selection forces. During this evolution process, renal cancer cells acquire ‘attributes’ that allow for an ability to resist exogenous growth-inhibitory signals, to evade apoptosis, to proliferate without limits (i.e., to undergo immortalisation), to thrive in a low-oxygen environment, to avoid immunosurveillance, to recruit angiogenic factors, to invade the basement membrane, and ultimately to spread to distant sites (Hahn and Weinberg, 2002). During this multistep process, the genome of the evolving cell acquires mutations within protooncogenes, tumour-suppressor genes, and other genes that regulate cell replication and growth (Loeb, 1991). Thus, oncogenesis can be viewed as an evolutionary process associated with multiple rounds of mutation, coupled with selection for specific ‘attributes’. Multiple lines of evidence, including conventional cytogenetic analysis, spectral karyotype studies, and comparative genome hybridisation analysis show that RCC cells exhibit extensive genetic heterogeneity (Pavlovich *et al*, 2003; Pavlovich and Schmidt, 2004). Identification of sentinel mutations driving carcinogenesis and malignant transformation is crucial for developing targeted therapeutic strategies.

RCC has several histologic types, each arising from distinct regions of the renal epithelia, caused by a distinct set of gene mutations and exhibiting a unique clinical course. The most common types of epithelial renal tumours include: clear-cell renal carcinoma (75%), type 1 (5%) and type 2 (10%) papillary, chromophobe (5%), and oncocytoma (5%) (Motzer *et al*, 1996). Both sporadic and inherited forms of clear-cell RCC are strongly associated with mutations in the von Hippel–Lindau (*VHL*) gene. In familial VHL-related RCC, the inheritance pattern is autosomal dominant (Latif *et al*, 1993). This pattern is consistent with the Knudson's ‘two hit’ model, which states that (if an inherited malignancy is dependent on inactivating mutation of a tumour-suppressor gene) one copy of the defective gene is inherited and the second copy is mutated during development/growth, subsequently leading to a more rapid oncogenic transformation (Knudson, 1971).

Other RCC histologies are also associated with specific mutations. For example, type 1 papillary RCC is characterised by mutations in the tyrosine kinase domain of c-Met oncogene, while mutations in the fumarate hydratase gene of the Krebs cycle have been found in hereditary leiomyomatosis RCC, an inherited form of type 2 papillary RCC. Birt–Hogg–Dubé syndrome, associated with loss of function mutation of *BHD* gene, predisposes affected individuals to chromophobe RCC and oncocytoma (for a review see Linehan *et al*, 2004). It is currently not known (and subject to intense study) how individual sets of gene changes lead to cancers only in specific cell and tissue types. Presumably, multiple other mutations and epigenetic changes function in concert with the above-mentioned sentinel mutations to drive the malignant phenotype. In this review, we focus on recent advancements in clear-cell RCC, discussing advancements in the understanding of its biology and subsequent development of effective targeted therapy.

Clear cell RCC is a highly vascular tumour arising from epithelial elements within proximal tubules of nephrons. An early event during the evolution of clear-cell RCC is loss of function mutation of the *VHL* gene (Latif *et al*, 1993). Individuals who inherit one defective copy of the *VHL* gene have a substantial risk for developing a variety of neoplasias, and in these patients the specific type of VHL mutation (e.g., types of missense, frameshift, deletion, etc) can influence the risk and type of tumour development (for review see Kim and Kaelin, 2004). Approximately 40% of patients with inherited VHL syndrome expire from complications of metastatic RCC. The renal tumours are of clear-cell histology, typically occur at a young age, and are characterised by the presence of multiple primary tumours and ‘premalignant’ cysts located in both kidneys. In contrast, patients with sporadic clear-cell RCC typically have a single primary lesion. Direct sequencing experiments form these sporadic tumour samples show up to 75% of these patients have biallelic loss of function mutation of *VHL* genes, and up to 20% exhibit expression inactivation by hypermethylation (Herman *et al*, 1994). Currently, it is unknown if prognosis in patients with sporadic RCC is related to the type of VHL mutation/alteration. Understanding the function of VHL has contributed to development of novel therapeutic strategies.

## VHL FUNCTION

**Figure 1:**
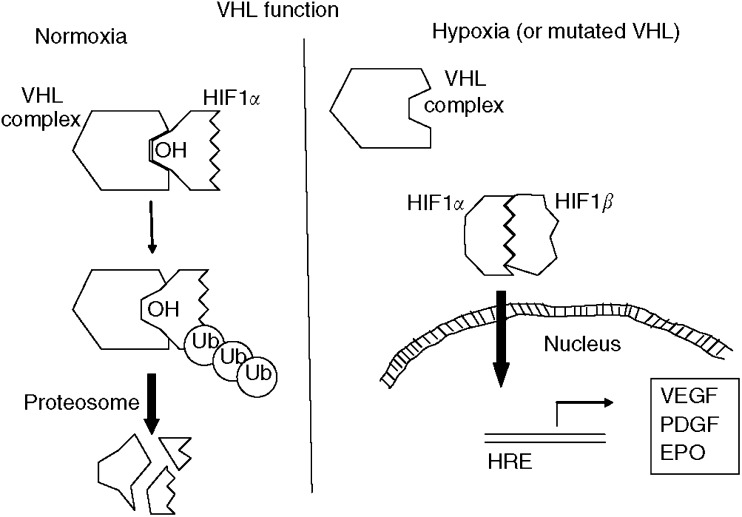
VHL and HIF-1 pathways. The VHL complex (composed of von Hippel–Lindau protein, elongin B, elongin C, Cul2, and Rbx1) functions to regulate levels of hypoxia-inducible factor (HIF)-1*α*. During normoxia, HIF-1*α* is hydroxylated at two proline residues via an oxygen-dependent enzymatic mechanism. The VHF complex binds to the hydroxylated HIF-1*α* and polyubiquinates HIF-1*α*, leading to proteosome-mediated degradation of HIF-1*α*. During hypoxia, HIF-1*α* is not hydroxylated, and thus cannot bind with the VHL complex. HIF-1*α* accumulates and binds to HIF-1*β*, thus forming the HIF-1 complex, which subsequently translocates into the cell nucleus where it binds with hypoxia-responsive element (HRE) in gene promotors and facilitates expression of hypoxia-inducible genes. Similarly, loss of function mutations of VHL prevents ubiquitin-mediated degradation of HIF-1*α*, resulting in upregulation of hypoxia-inducible genes.

The *VHL* gene is located on chromosome 3p25–26 (Latif *et al*, 1993), and functions in the hypoxia-inducible pathway (Figure 1). Early investigations showed the *VHL* gene product is found in a multiprotein complex composed of Elongin B, Elongin C, Cul2, and Rbx1 (Kamura *et al*, 1999). Sequence comparison showed that Elongin C and Cul2 is homologous to yeast proteins Skp1 and Cdc53, which form the so-called SCF complex (Skp1, Cdc53, and F-box protein). This complex of proteins function in yeast in ubiquitination, a process that ‘marks’ the targeted protein for degradation by proteosomes (Kamura *et al*, 1999). Biochemically purified VHL complex was subsequently shown to have ubiquitination activity. Specifically, the VHL complex ubiquitinates transcriptional factor hypoxia-inducible factor (HIF)-1*α* (Kamura *et al*, 2000). The normal function of HIF-1 complex (a heterodimer composed of *α* and *β* subunits) is to regulate expression of several genes in response to hypoxic stress (Wang and Semenza, 1993).

**Figure 2:**
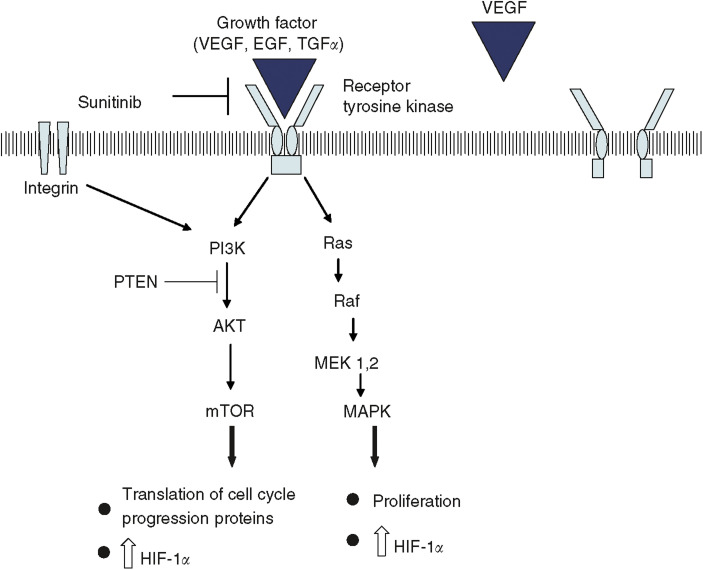
Overview of signal transduction pathways and role of selective inhibitors. Binding of a ligand (e.g., VEGF) to two adjacent receptors results in an active tyrosine kinase (e.g., VEGFR). The receptor tryosine kinase initially undergoes self-phosphorylation at specific tyrosine residues; this results in stimulation of several pathways. For example, RTKs can stimulate the Ras/Raf/MEK pathway, as the phosphotyrosines of RTKs facilitate docking of Grb2–SOS complex, ultimately resulting in activation of Ras. The activated Ras binds to Raf-1; afterwards, Raf-1 is activated via a complex series of phosphorylation and dephosphorylation steps. Ultimately, this pathway regulates expression of genes controlling apoptosis and cell proliferation. Similarly, mTOR is stimulated by a phosphorylation cascade, which involves proteins including PI3K and AK2. Once stimulated, mTOR controls protein translation of elements involved in cell cycle progression; in addition mTOR also controls protein synthesis in response to environmental change and starvation (including synthesis of HIF-1*α* in RCC cells). The signal transduction pathways can be inhibited at several steps including: (1) inhibition of VEGF (by bevacizumab); (2) inhibition of tyrosine kinase activity of RTK (by sunitinib and sorafenib); (3) inhibition of Raf kinase (by sorafenib); (4) inhibition of mTOR (by CCI-779).

Human cells respond to hypoxic conditions through a series of pathways, many of which are mediated by HIF-1 (Figure 1). Under normal conditions (i.e., with wild-type VHL and normal oxygen tension), HIF-1*α* is enzymatically hydroxylated at two proline residues located in the ‘oxygen-dependent degradation domain’. X-ray crystallography studies with VHL complexed with HIF-1*α* confirm this hydroxylation allows for hydrogen bond-mediated complex formation between the two proteins (Hon *et al*, 2002). HIF-1*α* is subsequently ubiquinated by the VHL complex and ultimately degraded within proteosomes. Under hypoxic conditions HIF-1*α* is not hydroxylated, and thus cannot bind and be efficiently ubiquitinated by the VHL protein complex. Biallelic inactivation of *VHL* would likewise prevent ubiquitination and ultimate degradation of HIF-1*α*. In addition to regulation by VHL complex, HIF-1 activity is regulated by growth factor and cell adhesion pathways. For example, upon binding of a growth factor to a tyrosine kinase receptor, HIF-1*α* protein levels increase through at least three pathways: (1) phosphatidylinositol 3-kinase-AKT-mammalian target of rapamycin (mTOR) pathway and (2) Ras/Raf/Map kinase pathway. Lastly, integration-mediated stimulation can also increase HIF-1*α* levels via PI3K/AKT-mTOR pathway (Figure 2; for a review see Bardos and Ashcroft, 2004).

Once stabilised, HIF-1*α* translocates into the nucleus where it complexes with the constitutively present HIF-1*β* to form the active transcriptional factor HIF-1 heterodimer. HIF-1 binds to a variety of additional transcriptional cofactors, forming a preinitiation complex of proteins that ultimately activates transcription of hypoxia-inducible genes including: vascular endothelial growth factor (VEGF; leading to angiogenesis; (Shweiki *et al*, 1992)), epidermal growth factor receptor (EGFR; leading to cell growth), platelet-derived growth factor (PDGF), glucose transporters (e.g., GLUT-1), transforming growth factor-*α* (TGF-*α*, ligand for EGFR) and erythropoietin (Bardos and Ashcroft, 2004). Many of these proteins are involved in angiogenesis, survival, pH regulation, and glucose metabolism. As mentioned above, the multistep model for carcinogenesis states that many of these properties are ‘acquired’ during tumour evolution (Loeb, 1991; Hahn and Weinberg, 2002). Phenotypically, RCC is a highly vascular tumour, with increased VEGF level, and its growth can be stimulated by factors produced through the HIF-1 pathway. This molecular biology understanding has facilitated the development of several therapeutic agents for RCC.

## SUNITINIB

SU11248 (Sunitinib) is an orally bioavailable small molecule that inhibits multiple split kinase domain receptor tyrosine kinases (RTKs) in tissue culture and *in vitro* experiments (including VEGF receptor 1 and 2, PDGF receptor *α* and *β*, KIT receptor, and FLT3 receptor (Mendel *et al*, 2003; Fabian *et al*, 2005)). A general model of how RTKs function includes: a growth factor first ligands to the extracellular portion of a specific RTK (Figure 2). The RTKs found at increased levels in RCC include: VEGF receptor, PDGF receptor, and EGF receptor, which ligand with VEGF, PDGF, and TGF-*α*, respectively. Subsequent to ligand binding, the receptor dimerises, and the intracellular C-terminal tyrosine residues of RTKs are phosphorylated. This step activates the kinase domain and initiates a signal transduction cascade that ultimately results in increased expression of several target genes (Figure 2; for a review see Krause and Van Etten, 2005). Inhibition of specific tyrosine kinases has been shown to be effective in treating several cancers that are ‘dependent’ on the TK pathway for proliferation/survival, including: chronic myelogenous leukaemia, gastrointestinal stromal tumours, breast cancer, and a subset of non-small-cell lung cancer. Sunitinib was created by rational drug design and inhibits tyrosine kinase activity of multiple split kinase domain RTKs by competitively binding with ATP at the tyrosine kinase active site.

In a phase I trial, sunitinib showed partial response to several tumours including RCC and gastrointestinal stromal tumours. In these initial studies, the agent was well tolerated, and fatigue was the dose-limiting side effect (Raymond *et al*, 2003). A regimen of 50 mg daily for 4 weeks, followed by 2 weeks off was recommended for phase II investigation (Table 1). In a phase II study with 63 cytokine-refractory metastatic RCC patients, 40% of patients had a partial response and 27% had stable disease for at least 3 months; the median time to progression was nearly 9 months (Motzer *et al*, 2005). Patients were also monitored for ‘molecular response’ by measuring biomarker levels of VEGF receptor inhibition, including plasma concentration of VEGF-A, VEGF-receptor 2, and PDGF. Interestingly, inhibition of the tyrosine kinase receptors resulted in decreased VEGF-receptor 2 levels during days 1–28 and concomitantly increased ligand levels during days of treatment (days 1–28), with some recovery of levels during the 2-week rest period. The clinical significance of this effect by the drug on the downstream elements of the VHL/HIF pathway is under investigation. Computed tomography imaging of responders shows findings consistent with necrosis, and it is likely that this agent has direct cytotoxic effects on tumour cells in addition to potentially inhibiting neovascularisation (Motzer *et al*, 2005).

A follow-up phase II trial completed accrual in 11/04 with 106 patients, and preliminary results are available (as of May 2005; Table 1). Notable difference between the two phase II trials: The second was limited to patients with clear-cell metastatic RCC, while the first trial included a small minority of patients with the papillary cell subtype. The overall response rate was similar, confirming the high response rate observed in the first trial. An international randomised phase III trial of sunitinib (administered in repeated cycles of 50 mg daily for 4 weeks then 2 weeks off) *vs* IFN-*α* (administered three times weekly) has recently completed accrual; interim analysis results will be available soon.

In the two phase II trials, sunitinib has been generally well tolerated, with compliance rate during the first 6 months of treatment of at least 95%; fatigue is the most common dose-limiting effect (incidence of grade 2–3 fatigue from the phase II trial is 38%). Other grade 2 or 3 side effects include diarrhoea (24%), nausea (19%), and stomatitis (19%). A rarer complication includes erythema is the soles of the feet and palms of the hands (8%); the pathophysiology of this side effect is currently under investigation.

## SORAFENIB

BAY 43–9006 (Sorafenib) is an orally bioavailable small molecule in the class of bis-aryl ureas that was initially found to potently inhibit the serine/threonine Raf-1 kinase (which phosphorylates proteins b-raf and c-raf); in tissue culture experiments, it has also subsequently been found to inhibit several RTKs including VEGF receptor-2 and VEGF receptor-3, FLT3 receptor, and PDGF receptor-*β* and c-KIT receptor (Wilhelm *et al*, 2004; Fabian *et al*, 2005). The Ras/Raf/MEK pathway leads to increased production of the HIF-1 complex; in addition, this pathway plays a central role in cell proliferation and apoptosis (Figure 2). The Ras/Raf/MEK pathway is initially activated following ligand binding with a RTK. This leads to a signal cascade which results in Ras activation, with subsequent formation of an active Ras/Raf protein complex; following propagation of this cascade, the final effect is upregulation of genes associated with cell proliferation and angiogenesis (Sebolt-Leopold and Herrera, 2004).

In a phase I trial, sorafenib had activity in a small sample of RCC patients (*n*=11) and a dose of 400 mg twice a day was found to be generally well tolerated (Strumberg *et al*, 2005). In a recently completed phase II study, 202 patients with metastatic clear-cell RCC were treated with sorafenib 400 mg b.i.d. for 12 weeks; afterwards, RCC patients with stable disease (<25% tumour shrinkage and <25% tumour growth) were randomised to either continue BAY43–9006 (*n*=32) or receive placebo (*n*=33). Following a 12-week induction, those with >25% tumour shrinkage continued on sorafenib as part of open-arm (*n*=73) and those with >25% growth (*n*=51) were taken off the study. Of the patients in the stable-disease arm who were randomised to receive sorafenib, the median progression-free survival was 24 weeks after randomisation, relative to 6 weeks in the placebo group (Ratain *et al*, 2005).

Currently, a randomised phase III trial comparing sorafenib *vs* placebo is under way involving approximately 900 patients with metastatic clear-cell RCC. A recent interim analysis (*n*=335 in sorafenib arm and *n*=337 in placebo arm) showed the median duration of progression-free survival was 24 weeks in patients treated with sorafenib compared to 12 weeks in the placebo group (Table 1). After at least 6 weeks of treatment, 79% of patients were progression free in the sorafenib arm (2% with partial response defined by RECIST criteria and 78% with stable disease, defined as between 25% reduction and 25% growth), compared to 55% in the placebo arm (0% with partial response and 55% with stable disease) (Escudier *et al*, 2005). The most common side effects included hand–foot skin reaction (26%), diarrhoea (30%), alopecia (23%), fatigue (18%), nausea (14%), and hypertension (8%) (Escudier *et al*, 2005; Ratain *et al*, 2005). Together, these data show that sorafenib shows efficacy in metastatic RCC. The majority of patients had stable disease with prolonged progression-free survival, albeit only 2% have a partial response based on RECIST criteria. Interestingly, the partial response rate of sorafenib is less relative to that of sunitinib, and the stable disease rate is similar. The relatively lower partial response rate of sorafenib may be related to the apparent binding affinity of RTKs including VEGF-receptor-2 and PDGF-receptor-*β* is weaker for sorafenib relative to sunitinib (Fabian *et al*, 2005).

## BEVACIZUMAB/ERLOTINIB

As discussed, RCC is a highly vascular tumour associated with high VEGF and EGF receptor levels. Early studies attempted to block tumour vasculature with bevacizumab (Avastin), a recombinant monoclonal antibody that binds VEGF, thus blocking its interaction with the VEGF receptor. A phase II trial comparing placebo (*n*=40), low dose (4.5 mg kg^−1^ loading dose and 3 mg kg^−1^ day 7 and every 2 weeks afterward; *n*=37) and high dose (15 mg kg^−1^ loading dose, 10 mg kg^−1^ on day 7 and every 2 weeks afterward; *n*=39) showed modest partial response (10%) with the high-dose regimen. Median time to progression was 2.5 months with placebo, 3 months with low dose, and 4.8 months with high-dose bevacizumab (Yang *et al*, 2003). As noted above, TGF-*α* and its receptor, EGFR, are frequently overexpressed in RCC. However, single-agent clinical trials in RCC patients with either monoclonal antibodies or single molecules that block EGFR tyrosine kinase activity (e.g., with erlotinib) have been disappointing.

A compelling approach is to combine agents that block important pathways (e.g., inhibit VEGF and EGFR) simultaneously. In a recently published phase II trial with bevacizumab (10 mg kg^−1^ every 2 weeks) and erlotinib (Tarceva; 150 mg daily), of the 59 evaluable clear-cell RCC patients, 3% had complete response, 22% partial response, and 61% stable disease following 8 weeks of treatment. The median time to progression was 11 months. The most significant reported side effects of the combined regimen included hypertension, proteinuria, diarrhoea, and acne-like rash (Hainsworth *et al*, 2005). A randomised phase II trial of bevacizumab alone *vs* bevacizumab and erlotinib recently completed accrual. The results are anticipated soon, and will assess the benefit of combination therapy *vs* bevacizumab alone. Interestingly, erlotinib was originally shown to be effective in patients with non—small-cell lung cancer; a large percentage of the patients who respond to erlotnib have an activating mutation within the EGFR tyrosine kinase domain (Pao *et al*, 2004; Tsao *et al*, 2005). It is currently unclear if RCC patients who respond to the combination of erlotinib and bevacizumab similarly have a gain-of-function mutation within the tyrosine kinase active site and/or if they simply overexpress EGFR/ligand or VEGF/receptor.

## CCI-779 (TEMSIROLIMUS)

Mammalian target of rapamycin is a serine/threonine kinase involved in the PI3K and AKT pathways (Figure 2). This pathway is activated upon binding of a growth factor (e.g., VEGF, PDGF) to a RTK. AKT is inhibited by the tumour-suppressor PTEN; hence this pathway is activated in tumour cells containing a mutated PTEN. A downstream effect of this pathway is mTOR phosphorylates a component of the mRNA translation initiation, resulting in increased translation of several proteins involved in cell cycle progression; in addition, there is increased translation of HIF-1*α*. CCI-779, a derivative of the immunosuppressive agent rapamycin, forms a complex with FK-506-binding protein-12, and this complex inhibits mTOR kinase activity (Meric-Bernstam and Mills, 2004).

Phase I study with CCI-779 showed reversible, tolerable side effects including acne, mucositis, hyperlipidaemia, asthenia, diarrhoea, and nausea. Interestingly, immune suppression was apparently not a major side effect at doses tested. In a phase II study involving 111 patients with refractory metastatic RCC, partial response was seen in 7% and clinical benefit rate (i.e., patients who either exhibited a complete response, a partial response, or had stable disease) for at least 24 weeks was 51%, with median time to progression of 5.8 months (Atkins *et al*, 2004).

A combination of CCI-779 and IFN*α* was tested in a phase I/II trial involving 71 patients with metastatic RCC. The maximum tolerated dose was CCI-779 15 mg IV weekly and IFN*α* 6 million units three times weekly. Of the 71 enrolled patients, partial response was seen in 11% and stable disease in 30%; median time to progression was 9.1 months (Smith *et al*, 2004). The most common side effects included: leukopenia (25%), hyperlipidaemia (15%), asthenia (13%), AST increase (8%), mucositis (6%), anaemia (6%), thrombocytopenia (6%), and rash (6%). A phase III clinical trial comparing CCI-779, IFN-*α*, or a combination of both is under way, with results pending.

## INSIGHTS FROM CLINICAL TRIAL RESULTS

Characterisation of the VHL and HIF pathways has led to a more detailed understanding of RCC biology, with subsequent development of effective therapy. Many VHL/hypoxia-induced regulated proteins are RTKs and their ligand; inhibition of RTK(s) shows promise in treatment of RCC. It is currently unclear whether the major effect of these agents is through inhibition of RTKs residing within the tumour cells or the surrounding stromal cells; thus it is unknown if these agents are directly cytotoxic to cancer cells, or rather these agents arrest proliferation of tumour stroma (e.g., by blocking angiogenesis). Our experience with sunitinib is that responding patients generally exhibit evidence of tumour necrosis within the first few treatment cycles; although this finding is consistent with significant direct tumoricidal effect, we do not yet know the contribution of angiogenesis blockade within these patients. It should be noted that because these therapeutic agents inhibit multiple RTKs and pathways, it is difficult to discern which receptor(s) are the most physiologically relevant in RCC. One hypothesis is RCC tumour (and surrounding stroma) proliferation is driven by stimulation of multiple RTK receptors/pathways; thus, an effective therapeutic agent must simultaneously inhibit multiple RTKs. Alternatively, perhaps the tumour cells and vascular endothelial cells are all stimulated by one set of ligand/receptor pair (i.e., VEGF and its receptor(s)). The most relevant RTK(s) for RCC growth will likely become more apparent over the next few years as results from clinical trails and laboratory experiments mature.

Unfortunately, the clinical response to these agents is not permanent; rather the time to progression is, on average, approximately 6–12 months. Tumours adapt; the genes and mutations responsible for this resistance are unknown. One thought is, since resistance occurs, these agents may not solely act on normal endothelial and stromal cells to block angiogenesis; rather these agents may act directly on the evolving cancer cells (e.g., to block proliferation signals), and over time, the tumours mutate and evolve. Consistent with this hypothesis, resistance to other RTK inhibitors in diverse tumours (e.g., imatinib in chronic myelogenous leukaemia and erlotinib in non-small-cell lung cancer) can occur via active site RTK mutations within tumour cells, such that the binding affinity of the inhibitor is diminished. Alternatively, assuming that the major action of these agents is blockage of angiogenesis, the cancer cells may adapt by simply releasing higher concentration of ‘growth factors’, sufficient to overcome RTK inhibition by these drugs. Comprehensive studies to address mechanism of resistance are required to reconcile between the competing theories.

## CONCLUSION AND FUTURE DIRECTIONS

Over the past 5 years, we have witnessed rapid advances in the understanding of RCC biology. This understanding has translated into development of therapies with improved clinical response. In phase II clinical trials these therapies have an overall response rate approaching 40% (Table 1); in contrast the current standard of care (IFN-*α* or IL-2) has a response rate of approximately 10–15%. We await the results of phase III trials to precisely define the role of the targeted agents in metastatic clear-cell RCC. Unfortunately, following targeted therapy, response is not permanent, and not all patients benefit clinically from these agents. In the near future, we will attempt to identify molecular markers that are associated with good clinical response. In addition, efforts will be made to determine the most effective dosing schemes. These agents will be given to patients with other forms of renal cancers, including papillary renal cell cancers, as part of a phase II clinical trial. In order to further enhance response rates, we look for combination treatment strategies that concurrently inhibit multiple growth factor pathways including RTKs (e.g., receptors for VEGF, PDGF, and EGF), phosphatidylinositol 3-kinase-AKT-mTOR, and Ras/Raf/Map kinase pathways. In addition, we look for the development of specific HIF-1 inhibitors; to our knowledge there are no reports of an effective HIF-1 inhibitor in animal models. Ultimately, we expect to learn that the evolution of RCC is not entirely dependent on HIF-1 and RTKs, and other pathways that drive clear-cell RCC progression will need to be discovered and novel inhibitors synthesised and tested clinically.

## Figures and Tables

**Table 1:** Clinical trials involving targeted agents for RCC

**Agent**	**No. of patients**	**Overall response^a^ (%)**	**Time to progression (months)**
Sunitinib			
Phase II	63	40	8.7
Sorafenib			
Phase III	335	2	6.0
Bevacizumab			
High-dose arm	39	10	4.8 (*P*<0.001 *vs* placebo)
Bevacizumab and erlotinib	59	25	11.1
CCI-779 and IFN*α*	71	11	9.1

a Summation of partial and complete response based on response evaluation criteria in solid tumours (RECIST).
